# Cyclodextrin-based nanosponges as drug carriers

**DOI:** 10.3762/bjoc.8.235

**Published:** 2012-11-29

**Authors:** Francesco Trotta, Marco Zanetti, Roberta Cavalli

**Affiliations:** 1 Dipartimento di Chimica. University of Torino, Via Pietro Giuria 7 10125 Torino, Italy; 2 Dipartimento di Scienza e Tecnologia del Farmaco. University of Torino, Via Pietro Giuria 9 10125 Torino, Italy

**Keywords:** controlled release, cross-linked polymers, cyclodextrin, drug delivery, nanosponges

## Abstract

Cyclodextrin-based nanosponges, which are proposed as a new nanosized delivery system, are innovative cross-linked cyclodextrin polymers nanostructured within a three-dimensional network. This type of cyclodextrin polymer can form porous insoluble nanoparticles with a crystalline or amorphous structure and spherical shape or swelling properties. The polarity and dimension of the polymer mesh can be easily tuned by varying the type of cross-linker and degree of cross-linking. Nanosponge functionalisation for site-specific targeting can be achieved by conjugating various ligands on their surface. They are a safe and biodegradable material with negligible toxicity on cell cultures and are well-tolerated after injection in mice. Cyclodextrin-based nanosponges can form complexes with different types of lipophilic or hydrophilic molecules. The release of the entrapped molecules can be varied by modifying the structure to achieve prolonged release kinetics or a faster release. The nanosponges could be used to improve the aqueous solubility of poorly water-soluble molecules, protect degradable substances, obtain sustained delivery systems or design innovative drug carriers for nanomedicine.

## Review

Recent advances in nanotechnology demonstrate the increased attention that is now being paid to the supramolecular assembly of simple components for therapeutic and diagnostic purposes. The design of new biomaterials based on nanoscale structural characteristics can be expected to provide many potential applications in the field of nanomedicine.

Cyclodextrins [1–3] are nanometric biomaterials with a close relationship between molecular status and supramolecular properties. They are a class of cyclic glucopyranose oligomers and are synthesised by enzymatic action on hydrolysed starch. The main common native cyclodextrins are α, β and γ, which comprise six, seven and eight glucopyranose units, respectively. They have a characteristic toroidal shape, which forms a well-defined truncated cone-shaped lipophilic cavity. Cyclodextrins are able to include compounds whose geometry and polarity are compatible with that of their cavity.

However, native cyclodextrins are not able to form inclusion complexes with certain molecules, such as hydrophilic or high-molecular-weight drugs. Moreover, β-cyclodextrin, the cheapest type, has low water solubility (1.85% w/v at 25 °C) and is toxic when injected intravenously. Consequently, many chemical modifications of cyclodextrins have been studied in an attempt to overcome their limitations and improve their technological characteristics. Well-structured molecules as well as random mixtures are described in the literature [4], and dimers [5], trimers [6], and polymers have also been obtained [7].

A different approach is to synthesise cross-linked cyclodextrin-based polymers so as to prepare insoluble multifunctional cyclodextrin derivatives [8–10]. These polymers can be obtained by reacting native cyclodextrins with a cross-linking agent that, after reaction, exerts its own properties and influences the behaviour of the cyclodextrin unit. Although insoluble cross-linked cyclodextrin polymers were first reported a long time ago, by reacting the parent cyclodextrin with dialdehydes, epoxides, epichlorohydrin, diacyl chlorides, etc., the term cyclodextrin nanosponges were first used by DeQuan Li and Min Ma in 1998 [11] to indicate a cross-linked β-cyclodextrin with organic diisocyanates leading to an insoluble network that showed a very high inclusion constant with several organic pollutants. For instance, *p*-chlorophenol was almost completely removed from waste water even at the parts per billion level [12]. However, no other applications were claimed or proposed.

It was not until the work performed by Trotta and co-workers [13] and the syntheses of new kinds of cyclodextrin nanosponges that they revealed their full potential in other fields, particularly as drug carriers.

**Scheme 1:**
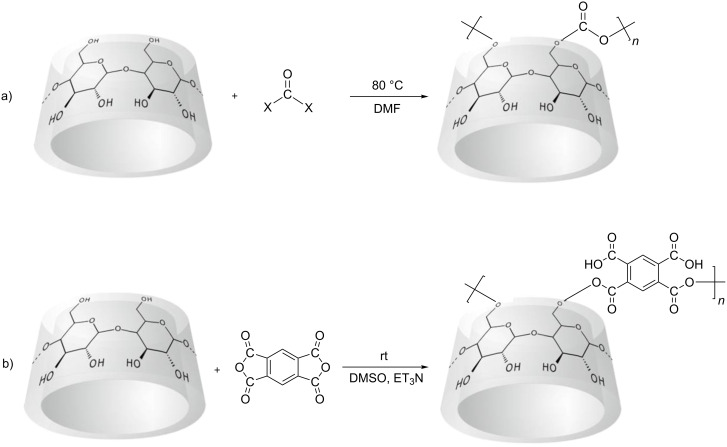
Synthetic routes to cyclodextrin nanosponges. (a) Cyclodextrin carbonate nanosponges. (b) Cyclodextrin carboxylate nanosponges.

Generally speaking, nanosponges are hyper-cross-linked cyclodextrins that can be obtained with α, β and γ cyclodextrins, either alone or as mixtures containing relevant amounts of linear dextrin, cross-linked with a suitable cross-linking agent. Interesting results have already been obtained as drug carriers by using an active carbonyl compound, e.g., carbonyldimiidazole, triphosgene, diphenyl carbonate, or organic dianhydrides (Scheme 1).

**Figure 1:**
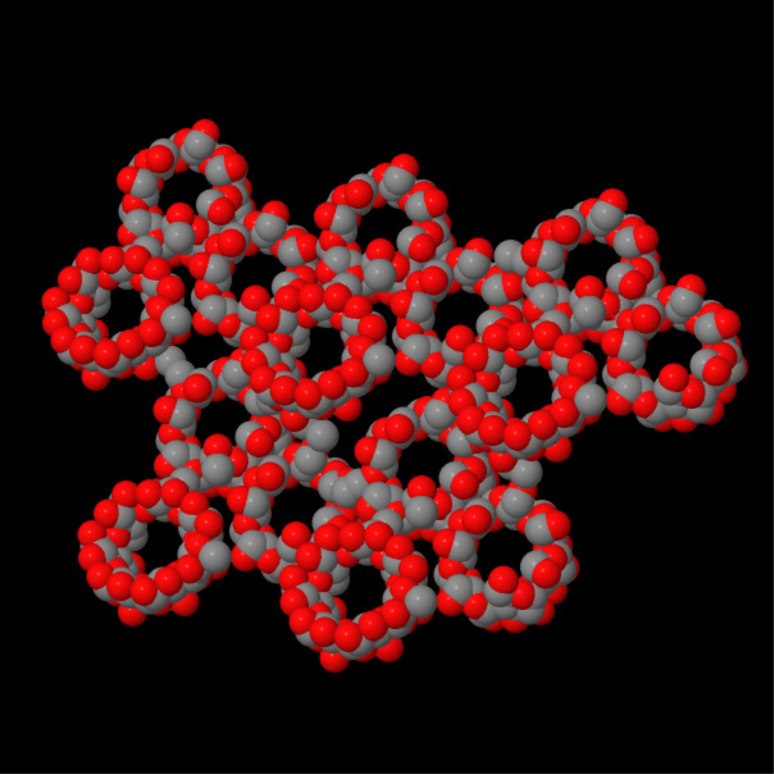
Molecular structure of cyclodextrin carbonate nanosponges.

Detailed reaction conditions and parameters are reported elsewhere [14]. Here we simply wish to stress that primary hydroxy groups are mainly involved in the formation of a network as shown by FTIR–ATR, Raman and solid-state NMR analyses [15]. Moreover, the elastic properties of cyclodextrin nanosponges were determined by analysis of the spectral modification of the Boson peak and Brillouin frequency [16]. Using ultrasound-assisted synthesis and a suitable cross-linker molar ratio, spherical nanosponges of submicron size were obtained [17]. The cross-linking produces a powder consisting of cyclodextrin connected by nanochannels to form a cage-like structure (Figure 1).

By using different amounts of cross-linking agent, or by changing the type of cyclodextrin, it is possible to modulate the channels between the cyclodextrin molecules, thereby tuning the formation of the porous network and consequently affecting both the inclusion capacity and the solubilising ability of the nanosponges. Acid nanosponges carrying free carboxylic groups can be obtained using pyromellitic anhydride or other dianhydrides as a cross-linker, forming true cation exchange sites. The presence of free hydroxy groups in the nanosponges network allows further surface modification. For instance, carboxylated nanosponges can be obtained by reacting pristine carbonate nanosponges with succinic anhydride.

Detailed physicochemical characterisation is reported in the literature, demonstrating that carbonate nanosponges are thermally stable up to 300 °C and can therefore be sterilised by autoclaving at 121 °C and 2 bar for 15 minutes. FTIR and NMR analyses before and after sterilisation show their stability and the absence of degradation [13]. In addition, they do not act as surfactants and can be easily dispersed in water.

Nanosponges also have colloidal sizes with a mean diameter of less than 1 µm and narrow size distribution and form opalescent suspensions on dispersion in water. The zeta potential of carbonate nanosponges is about −25 mV, which is sufficiently high to produce stable water suspensions that do not undergo aggregation over time.

**Figure 2:**
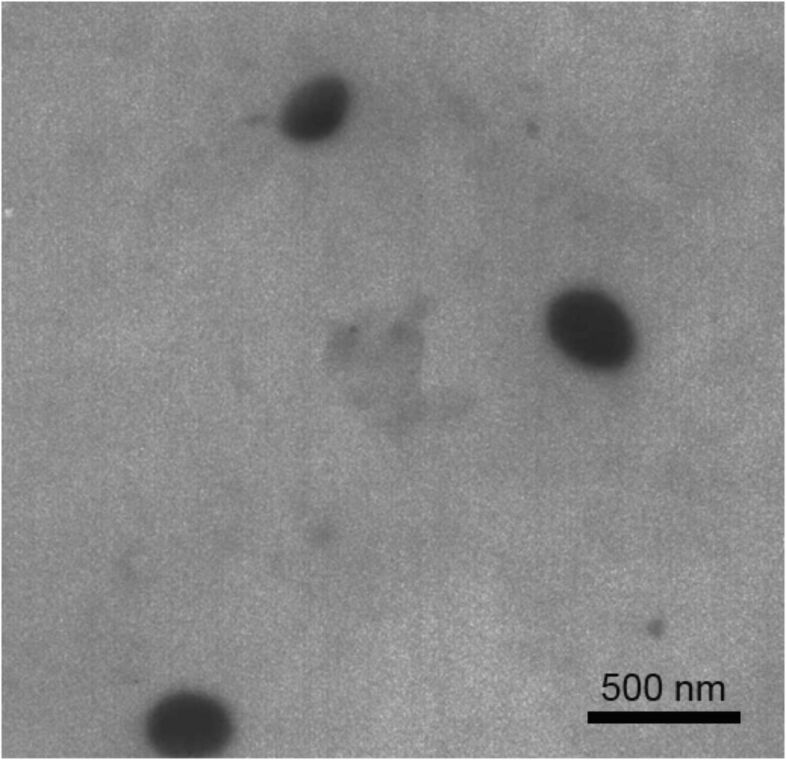
TEM microphotograph of cyclodextrin carbonate nanosponge (magnification 46,000×).

For parenteral application it is possible to synthesise nanosponges with a mean diameter in the range of 200–300 nm with narrow size distribution (Figure 2).

The stability of carbonate nanosponges was evaluated at 60 °C under both acidic and basic conditions. Under acidic conditions (0.1 N HCl) a limited release of cyclodextrin units was observed after 2 h due to degradation of the nanosponge structure, whereas the basic environment did not affect the nanosponge stability. On the other hand, carboxylated nanosponges obtained by using pyromellitic dianhydride as a cross-linker appear less chemically and thermally stable, but can swell more than 30-fold in water, they have more negative zeta potential, and can simultaneously host cations, organic molecules and even macromolecules.

Preliminary toxicity studies were carried out on carbonate nanosponges in order to assess their safety with respect to pre-clinical studies on laboratory animals.

The methods employed for cytotoxicity testing are those described in the literature [18].

In vitro cell culture toxicity assays were carried out on different cell lines (i.e., HELA; MCF7, COS) by using the MTT test. Cells were incubated for between 24 and 72 h and their viability determined. After incubation with nanosponges no cytotoxicity was observed. The percentage of cell viability did not change from untreated cells. Other in vitro methods for evaluating the toxicity of new materials include the determination of its haemolytic properties. When nanosponges were incubated with human erythrocytes for 90 minutes, no haemolytic activity was observed up to a concentration of 15 mg/mL, showing that the nanosponges possess good blood compatibility.

Acute systemic toxicity was evaluated after injection in mice. Unlike other cross-linked cyclodextrin polymers [19], nanosponges were found to be safe between 500 and 5000 mg/kg in Swiss albino mice: they did not show any sign of toxicity or adverse reactions. Nanosponges were then injected intravenously into mice and monitored for 24 h. Oral administration of nanosponges was also tested in mice [20] with no apparent side effects.

The nanoporous structure that is formed changes many properties of the native cyclodextrin. This peculiar and versatile structure is suitable for a broad range of potential and actual applications and was recently reviewed [13,21–23]. As a general characteristic, the nanosponge structure improves the ability of cyclodextrins to form specific complexes with guest molecules, compared to native cyclodextrins. The included substances can be either strongly retained or released in a controlled manner.

From an environmental standpoint this means that water pollutants, such as heavy metals and many organic molecules, can be removed and retained easily [24–25].

The use of nanosponges in water treatment has been found successful for aromatic chlorohydrocarbons, and nanosponges succeeded where their main competitor, activated carbon, failed, for example, in the removal of heavy metals. Furthermore, nanosponges have greater mechanical strength than activated carbon and are not affected by dust formation during application. Finally, they can be reused indefinitely after simple washing with an environmentally friendly solvent, such as ethanol.

Nanosponges can be used as supports for catalysis applications. One important result that has emerged from experiments on enzyme catalysis is the prolonged activity of the enzymes [26]. Nanosponges can preserve the activity of some enzymes far longer than other supporting media, such as agarose. Moreover, the possibility of including enzymes inside the structure prolongs their activity and makes it possible to obtain an efficaciously supported catalyst to promote chemical reactions and aid the destruction of chemical pollutants (detergents, POPs abatement) as well as biosensors.

Nanosponges can also be used in agriculture and have shown the interesting characteristic of encapsulating some important agricultural chemicals. In this case, release is controlled and has a slow profile [27]. Finally, nanosponges can also be used in polymer processing to improve flame-retardant properties [28–29] and in enantio-differentiating photoisomerization reactions [30].

### Application of cyclodextrin nanosponges in drug delivery

By virtue of their biocompatibility and versatility, nanosponges have many potential applications in the pharmaceutical field. One recent EU commission report focused on the use of cyclodextrin nanosponges as a promising innovative system for drug delivery. They can be used as excipients in preparing tablets, capsules, pellets, granules, suspensions, solid dispersions, or topical dosage forms or as new nanotechnological, multifunctional carriers [31–32].

In particular, the design of new nanodelivery systems to improve drug administration is currently being studied. Many drugs show poor solubility, low permeability, a short half-life, low stability, and/or high molecular weight, and as a consequence their formulation is challenging.

Nanosponges have the capacity to incorporate drugs within their structure, either as inclusion complexes or as noninclusion complexes. There are far more interaction sites available for incorporation within a nanosponge structure than in β-cyclodextrin molecules, and they also show different mesh polarities (hydrophobic cyclodextrin cavities surrounded by hydrophilic nanochannels of the polymeric network) thereby enabling significant interactions between molecules with different lipophilicities and structures.

The ability of cyclodextrin nanosponges to encapsulate drugs has been studied in depth for both hydrophilic and lipophilic molecules, including dexamethasone, flurbiprofen, doxorubicin, itraconazole, resveratrol, paclitaxel, 5-fluorouracil and tamoxifen, as is shown in Table 1.

**Table 1:** Molecules complexed by using nanosponges.

Drug	log P	Therapeutic activity	Administration route	Reference

Dexamethasone	1.9	anti-inflammatory	oral, parenteral	[51]
Flurbiprofen	4.2	anti-inflammatory	oral	[51]
Doxorubicin	1.3	antineoplastic	parenteral	[51]
Progesterone	3.9	hormonal	oral	[51]
Itraconazole	5.7	antifungal	oral, topical	[33]
5-fluorouracile	−0.9	antineoplastic	parenteral, topical	[40]
Tamoxifen	4.0	antiestrogen	oral	[34]
Resveratrol	2.8	antioxidant	oral, topical	[48]
Paclitaxel	2.5	antineoplastic	parenteral	[36–46]
Camptothecin	1	antineoplastic	parenteral	[41–42]
Omeprazole	2.2	antiulcerative	oral	[38]
Nelfinavir mesylate	4.6	antiviral	oral	[38]
Acetylsalicylic acid	1.2	analgesic	oral	[36]
Acyclovir	−1.6	antiviral	oral, topical, parenteral	[39]
Gamma-oryizanol	–	antioxidant	topical	[49]
Telmisartan	7.7	antihypertensive	oral	[47]

Considering their versatility, nanosponges can act as multifunctional carriers, i.e., to enhance solubility, protect fragile molecules, and achieve sustained release. In many cases they act with two or more functions simultaneously.

In this review we summarise their potential capacities by classifying nanosponges according to their main applications, and some examples are reported as case studies.

### Solubility enhancement

One of the greatest limits to the development of various pharmaceuticals is the low water solubility of many drugs. About 40% of new drugs are poorly soluble in water, which hinders their clinical application. The formulation of poorly water-soluble drugs constitutes a problem that is difficult to solve. Many technological approaches have been investigated. The ability of cyclodextrins to form inclusion complexes with various molecules is widely used in the pharmaceutical field as a strategy to increase the aqueous solubility and, consequently, bioavailability of lipophilic drugs. For hydrophilic or moderately polar drugs this approach is less effective, and consequently cyclodextrin derivatives have been investigated.

Nanosponges can improve the wetting and solubility of molecules with very poor solubility in water. The drugs can be molecularly dispersed within the nanosponge structure and then released as molecules, avoiding the dissolution step. Consequently, the apparent solubility of the drug can be increased.

Many formulation and bioavailability problems can be solved by enhancing the solubility and dissolution rate of a substance, and nanosponges can greatly enhance the drug solubility.

Swaminathan et al. studied a formulation of itraconazole in nanosponges [33], a drug with an aqueous solubility of about 1 ng/mL at physiological pH. The presence of nanosponges improved the solubility of the drug more than 27-fold; and when PVP was added as an auxiliary component of the nanosponge formulation, this rose to 55-fold. Moreover, the dissolution profiles of the drug for the two formulations were faster than for the formulations available on the market. Nanosponge formulation could thus increase the bioavailability of itraconazole.

The solubilisation efficiency of nanosponges with regard to tamoxifen, a nonsteroidal anti-estrogen molecule used for treating and preventing breast cancer, was observed by using a 0.5% aqueous suspension of β-CD nanosponges [34]. Encapsulation of tamoxifen in these nanosponges was more than 40% w/w, since 5 mg of nanosponges can solubilise 2.2 mg of tamoxifen. The in vitro release of tamoxifen from nanosponges showed pseudo-zero order kinetics. After 2 h about 60% of the encapsulated drug had been released at pH 7.4.

The anticancer agent paclitaxel is another drug difficult to formulate. Paclitaxel has a very low solubility in water, i.e., less than 0.3 μg/mL. As a consequence, the dosage form available for clinical administration requires the use of other pharmaceutical solvents or emulsifying agents, namely Cremophor EL (polyethoxylated castor oil). Unfortunately, Cremophor EL has been seen to cause serious side effects, such as neurotoxicity, nephrotoxicity and cardiotoxicity [35].

Cyclodextrin-based nanosponges were used to prepare a Cremophor-free formulation for paclitaxel administration. Paclitaxel-loaded nanosponges were prepared by using the freeze-drying method to entrap the drug in a β-CD nanosponges network. The paclitaxel solubilisation efficiency of this formulation was compared to that of native β-cyclodextrin showing a marked increase. β-CD nanosponges loaded with paclitaxel have a mean diameter of about 350 nm with a rather narrow size distribution (polydispersity index below 0.2).

These nanosponges obtained using carbonyldiimidazole as a cross-linker showed good paclitaxel complexation ability: one millilitre of a 1.5% nanosponge aqueous suspension allows one to dissolve about 2 mg of paclitaxel, thus confirming their wetting and solubilising properties towards paclitaxel avoiding the use of surfactants. The paclitaxel-loaded nanosponges formed a stable colloidal system in water inhibiting the recrystallization of paclitaxel over time. Moreover, this study demonstrated that delivery of paclitaxel via nanosponges increased the amount of paclitaxel entering cancer cells and lowered the IC_50_ of paclitaxel, thereby enhancing its pharmacological effect [36].

### Sustained delivery system

Modified-release dosage forms offer a number of advantages over the conventional release formulation of a drug. The design of a modified-release product is generally intended to optimise the treatment regimen by providing slow, continuous delivery of the drug over the entire dosing interval. This makes it possible to decrease the dose administered, change the pharmacokinetic profile, and decrease side effects.

Different drug delivery systems have been designed to modify the release kinetics of medicinal products. The drug release kinetics from nanosponges can be obtained with a prolonged release profile over time. Previous in vitro studies showed that flurbiprofen was released slowly from β-CD nanosponges [37], reaching a percentage of less than 10% after 130 minutes. A similar result was obtained with the incorporation of doxorubicin in nanosponges. In in vitro tests, doxorubicin was released very slowly at pH 1.2 (about 1% after 120 minutes) and the percentage increased with pH values. Doxorubicin release of about 29% was obtained at pH 7.4. This behaviour could suggest that the nanosponge formulation is able to protect the drug from the gastric environment, allowing delivery in the intestinal tract.

Vavia prepared nelfinavir mesylate loaded nanosponges to enhance the solubility of the drug [38]. Nelfinavir is a protease inhibitor with low bioavailability, used to treat HIV infections. In this case, the drug was released more slowly from nanosponges than from a β-CD complex. This behaviour shows that nanosponges are able to prolong drug release over time and consequently could also be proposed as a sustained drug-delivery system for oral administration.

Acyclovir is a medium polarity drug with a solubility in water of 1.5 mg/mL. Special carboxylated nanosponges, containing dissociable carboxylic groups in their structure were developed for its encapsulation. They represent a further electrostatic contribution for drug encapsulation, in addition to the cyclodextrin cavities. Electrostatic interactions may occur between the carboxylic groups present in the nanosponge structure and the amino group of acyclovir.

In vitro, acyclovir-loaded carboxylated nanosponges [39] showed prolonged release of the drug without the initial burst effect, and 20% drug release was obtained after three hours.

### Protection from light or degradation

Nanosponges can also be used as carriers to protect encapsulated molecules from light or from chemical- and enzyme-induced degradation. To evaluate the potential protection application, 5-fluorouracile was used as a light-sensitive model drug. β-CD nanosponges were able to incorporate up to 30% of 5-fluorouracile [40]. The in vitro release of 5-fluorouracile, determined by using the dialysis-bag technique at pH 7.4, was about 60% of the encapsulated amount after 2 h showing an interaction between the drug and the nanosponge structure, despite the hydrophilicity of the drug. Moreover, encapsulation of 5-fluorouracile in nanosponges protected the drug and maintained its cytotoxicity against MCF-7 cells. Another paradigmatic example was established with the incorporation of camptothecin in cyclodextrin nanosponges.

The anti-tumour activity of camptothecin has been extensively investigated in both hematological and solid malignancies; however, its use is still limited due to its poor solubility and high chemical instability since the lactone ring of the molecule is very susceptible to hydrolysis under physiological conditions.

The encapsulation of camptothecin in nanosponges was used to prolong the shelf life and release of the drug [41]. The nanosponges solubilised large amounts of the drug and protected the lactone ring from opening due to its high inclusion abilities, thereby increasing stability.

Three types of nanosponges, i.e., β-CD-1/2 nanosponges, β-CD-1/4 nanosponges and β-CD-1/8 nanosponges, characterised by an increasing cross-linker/β-CD molar ratio, have been used as delivery systems for camptothecin. All proved to solubilise camptothecin, thereby increasing its aqueous solubility, and formed inclusion complexes. FTIR, DSC and XRPD analyses confirmed the drug interaction with nanosponges. Moreover camptothecin loaded in nanosponges was seen to be more effective than the plain drug on HT29 cells.

The same formulation was recently shown to be an effective nanotechnology for the treatment of both androgen-sensitive and castrate-refractory prostate cancer in cell-line experiments [42].

Nanosponges can be used to store and prolong the release of volatile molecules, such as essential oils, following their encapsulation. Linalool, a liquid component of many essential oils and fragrances with a boiling point of 198 °C, was encapsulated in different types of nanosponges as a liquid oil model [43]. β-CD-1/4 nanosponges can incorporate 8% w/w of linalool within their structure. The entrapment of linalool in the nanosponge matrix was confirmed by DSC analysis. In vitro release studies were carried out by using the linalool β-cyclodextrin complex for comparative purposes. After 2 hours, linalool release from nanosponges was half that from the β-cyclodextrin complex, thereby showing that nanosponges stabilise the molecule in their structure.

### Protein delivery

Swellable cyclodextrin-based nanosponges were purposely prepared for protein delivery by using a different synthetic route. New swellable cyclodextrin-based poly(amidoamine) nanosponges (PAA-NS) [44], named NS 10 and NS 11, were synthesised by cross-linking β-cyclodextrins with either 2,2-bis(acrylamidoacetic acid) or a short polyamido-amine chain deriving from 2,2-bis(acrylamidoacetic acid) and 2-methylpiperazine, respectively. These swellable nanosponges were shown to be sensitive to the pH of the surrounding media. PAA-NS were reduced in nanosuspensions by using the high-pressure-homoginisation technique. Bovine serum albumin (BSA) was used as a model protein to study the encapsulating capacity of these new β-cyclodextrin-based nanosponges. High protein-complexation capacity was achieved for both the nanosponges with a BSA-encapsulation efficiency greater than 90%. In vitro release studies were carried out, showing a prolonged release of albumin from the two swellable β-CD-nanosponges over a period of 24 h. The encapsulation of albumin and its prolonged release was also obtained with carbonate nanosponges (unpublished data).

NS10 and NS11 were recently used to incorporate lysozyme and showed a high loading capacity. The encapsulation efficiency and loading percentages were 89% and 17% for NS10 and 96% and 19.6 for NS11. These lysozyme-loaded nanosponges released the enzyme at a pH-dependent rate with prolonged kinetics, whilst maintaining its biological activity [44].

### Oral delivery systems

The dissolution rate of a solid drug is a limiting factor for oral bioavailability. For hydrophobic drugs the dissolution process acts as the rate-controlling step and, therefore, determines the rate and degree of absorption. As a consequence, many hydrophobic drugs show erratic and incomplete absorption from the gastrointestinal tract.

The Biopharmaceutics Classification System (BCS) was developed by Amidon [45] in 1995 as a tool for predicting the extent of drug absorption after oral administration. This system divides drugs into four categories according to their solubility and intestinal permeability. Formulation strategies can be used to shift a drug from one class to another by improving their pharmaceutical characteristics.

Acetylsalicylic acid (ASA), a nonsteroidal anti-inflammatory drug belonging to BCS class III, was formulated into pyromellitic dianhydride cross-linked β-cyclodextrin nanosponges [36]. TEM studies showed that the particle sizes of ASA-loaded nanosponges have average diameters ranging from 40 to 60 nm and they were seen to have a regular spherical shape. Zeta potential was high enough to obtain a stable colloidal formulation. In vitro and in vivo studies indicated a slow and prolonged ASA release from pyromellitic cross-linked β-cyclodextrin nanosponges over a long period, i.e., 24 hours. In carrageenan-induced rat paw edema, administration of ASA as a nanosponge formulation administered by oral gavage reduced inflammation significantly (P < 0.01 and P < 0.05) compared to plain ASA and the control group. These results indicate that the ASA nanosponge formulation may be used for oral delivery of the drug.

Paclitaxel-loaded nanosponges were administered to rats by oral gavage using commercially available TAXOL^®^ as the control. The oral bioavailability of the drug was increased about 3-fold after the administration of paclitaxel-loaded nanosponges, in comparison to the control [46].

More recently, Rao et al. studied the influence of carbonate nanosponges on telmisartan, an antihypertensive BCS class II drug characterised by an estimated solubility in water of just 9.9 μg/ml resulting in low bioavailability. The formation of a ternary complex of telmisartan with nanosponges and NaHCO_3_ was seen to synergistically enhance the dissolution rate of telmisartan [47].

### Topical delivery systems

Nanosponges can be used in gels or creams for topical application. In one recent work, resveratrol, a polyphenolic phytoalexin present in different plant sources and which plays an important role in the prevention of many human diseases, particularly due to its antioxidant properties, was encapsulated in nanosponges. Incorporation markedly increased the solubility and stability of the molecule.

Resveratrol-loaded nanosponges were seen to enhance drug permeation in in vitro studies on porcine skin. Enhanced permeation of resveratrol was also observed by using rabbit buccal mucosa [48].

Gamma-oryzanol is a ferulic acid ester mixture, used as sunscreen in the cosmetics industry. Its application is limited by its high instability and photodegradation. Gamma-oryzanol was encapsulated in nanosponges, showing a good protection from photodegradation. A gel and an O/W emulsion were formulated with the gamma-oryzanol-loaded nanosponges [49]. In vitro permeability and accumulation studies were then carried out on porcine skin. A high skin accumulation of gamma-oryzanol was observed over time. The ability of nanosponges to increase the uptake of the guest molecule by the skin can be attributed to the capacity to increase solubility at the surface of the skin, as already reported for cyclodextrins.

### Gas delivery

Gas storage and delivery play an important role in biology, medicine, cosmetics and pharmaceuticals. The molecular encapsulation of gases in cyclodextrin cavities has already been proven. Nanosponge formulations can act as a reservoir for various types of gas. β-CD nanosponges have shown an ability to store large amounts of carbon dioxide, 1-methylcyclopropene and oxygen [8].

Three different cyclodextrin nanosponges were synthesised cross-linking α-, β- or γ-cyclodextrin with carbonyldiimidazole as oxygen-encapsulating formulations [50]. The nanosponges were able to release oxygen both in the presence and in the absence of ultrasound (US). Oxygen permeation through a silicone membrane was obtained by using a nanosponge/hydrogel combination system

All types of nanosponges were able to encapsulate, store and release oxygen for prolonged periods. Ultrasound enhanced the in vitro release and permeation of oxygen. The nanosponge/hydrogel system produces a slower sustained release of the gas. Therefore, nanosponges could be suitable carriers for topical oxygen delivery in the presence and in the absence of US and could act as an oxygen reservoir.

## Conclusion

Nanosponges are a new type of biocompatible cross-linked polymer, whose production is flexible and cost-effective. Cyclodextrin nanosponges possess particular properties in terms of their encapsulation ability, biocompatibility and solubilisation capacity with regard to different types of molecules. Nanosponges could broaden the range of applications of cyclodextrins in pharmacy and medicine, as well as in other important fields, such as agriculture, cosmetics and the environment. They could be used to delivery two active substances simultaneously for combination therapy, or for simultaneous therapeutic and diagnostic applications. The surface engineering of nanosponges could lead to prolonged residence time in the blood or increased efficiency and specificity with ligands for targeting sites. In conclusion, nanosponges can be considered as multifunctional nanoscale systems suitable for the delivery of active molecules in nanomedicine.
